# Characteristics of Current Teaching Kitchens: Findings from Recent Surveys of the Teaching Kitchen Collaborative

**DOI:** 10.3390/nu15204326

**Published:** 2023-10-10

**Authors:** Christina Badaracco, Olivia W. Thomas, Jennifer Massa, Rachel Bartlett, David M. Eisenberg

**Affiliations:** 1 Avalere Health, 1201 New York Ave. NW Suite 1000, Washington, DC 20036, USA; 2 Nourishing Our Community, Boston Medical Center, 840 Harrison Ave., Boston, MA 02118, USA; 3 Department of Nutrition, Harvard T.H. Chan School of Public Health, 665 Huntington Ave., Boston, MA 02115, USAdeisenbe@hsph.harvard.edu (D.M.E.); 4 The Teaching Kitchen Collaborative, 101 Middlesex Turnpike, Suite 6, Burlington, MA 01803, USA; rachel.bartlett@teachingkitchens.org

**Keywords:** culinary medicine, interdisciplinary collaboration, teaching kitchens

## Abstract

Teaching kitchens are physical and virtual forums that foster practical life skills through participation in experiential education. Given the well-supported connection between healthy eating patterns and the prevention and management of chronic diseases, both private and public organizations are building teaching kitchens (TKs) to enhance the health and wellness of patients, staff, youth, and the general community. Although implementation of TKs is becoming more common, best practices for starting and operating programs are limited. The present study aims to describe key components and professionals required for TK operations. Surveys were administered to Teaching Kitchen Collaborative (TKC) members and questions reflected seven primary areas of inquiry: (1) TK setting(s), (2) audiences served, (3) TK model(s), (4) key lines of operations, (5) team member who manages or directs the TK, (6) team member(s) who performs key operations and other professionals or partnerships that may be needed, and (7) the primary funding source(s) to build and operate the TK (among various other topics). Findings were used to articulate recommendations for organizations seeking to establish a successful TK as well as for TKs to expand their collective reach, research capacity, and impact.

## 1. Introduction

The continued rise of diet-related diseases around the world indicates the need for nutrition interventions that are more effective in motivating and sustaining changes in dietary behaviors across various subpopulations and in real-life settings [1]. To meet this need, the field of culinary medicine, also referred to as culinary nutrition, has arisen over the past few decades to help translate nutrition education into practical skills—such as meal preparation and safe food handling—that can be used to improve physical and mental health [2]. Opportunities for culinary medicine to improve chronic disease management and prevention have been described previously [3].

Teaching kitchens (TKs) offer learning labs to translate these skills into practice and sustainable lifestyle change [4,5]. In addition to building cooking skills, they may also provide nutrition education, mindfulness instruction, exercise prescriptions, motivational interviewing, and other services [4]. They can lead to other positive outcomes such as building community, enhancing food security and sovereignty, improving health, reducing purchases and consumption of processed foods, etc. [6]. Importantly, they can also educate and empower participants of all ages—spanning from children to older adults—as well as families, thus benefiting multiple generations within one program [7,8]. These benefits can apply to audiences with various health conditions, from supporting patients through cancer treatment to teaching children basic cooking skills to encourage healthy diets from a young age [9,10]. Exemplifying the prominent body of literature proving their benefits, the 2023 Teaching Kitchen Research Conference featured more than 80 research posters that were presented by researchers from around the world [11].

TKs can also provide other services, such as a food pantry or healthy food prescription program [12,13]. Indeed, the increased interest in the field of culinary medicine is driven by many factors, such as the growth of the food is medicine (FIM) movement, increasing opportunities for health plans to cover food- and nutrition-related benefits, and the emerging body of literature in peer-reviewed journals on FIM-related studies that indicate improved patient health outcomes and potential for reduced healthcare costs. A recent resource developed by the Nutrition and Obesity Policy Research and Evaluation Network, which summarizes ways in which healthcare and public health can coordinate to enhance food and nutrition security in their communities, displays how food and nutrition interventions can be tailored to meet the needs of patients and communities across the continuum of health status, with the interventions for individuals who have capacity to cook their own foods forming the base of the pyramid of interventions and being appropriate for everyone—while also incurring the lowest cost [14].

Prominent organizations across the world are using TKs as catalysts of enhanced personal and public health across settings spanning healthcare institutions, community organizations, K–12 schools, universities and other academic settings, workplaces, and more. In addition to delivering programming to their respective audiences, many are engaged in program evaluation and dissemination of findings to support program improvement and broader implementation. Many such organizations are also working collaboratively in networks such as the Teaching Kitchen Collaborative (TKC) to develop new tools, engage in advocacy related to food and healthcare policy, conduct research to generate evidence, train future culinary medicine professionals, disseminate findings, and more.

Despite their growth, TK operations and impacts have not yet been described in the literature. As exemplified through the literature cited above, they vary widely in their structure and operations through the following characteristics: TK setting, audiences served, type(s) of TK, key lines of operations, team member who manages or directs the TK, team member(s) who performs key operations and other professionals or partnerships that may be needed, and the primary funding source to build and operate the TK (among various other topics). The TKC offers a large, diverse sample of organizations with TKs that track and provide detailed information through applications and/or membership surveys. This rich data source can be used to describe the individual and collective impact of a prominent sample of TKs as well as track changes over time and identify remaining gaps that need to be filled to optimize their impacts. Thus, the present study aims to describe the characteristics of representative TKs and their programs and to assess and summarize emerging opportunities for collective impact.

## 2. Materials and Methods

Data used to describe TKs in this study were derived from two surveys that were administered electronically to TKC members. Findings from the first survey were presented internally to staff and members at the 2019 TKC annual meeting, but have not yet been published.

The first TKC membership survey was initiated in 2017, two years after the inception of the organization. The goals of conducting this survey were to measure the collective impact of the collaborative; establish a baseline description of members/programs and capture their best practices to use for internal tracking, share with collaborative members, and publish research briefs; and gather data to inform strategic planning and refine the membership value proposition. The TKC intended to conduct the survey periodically in the future to track progress over time, as well.

Survey questions were written de novo with these goals in mind. Multiple choice question responses were pre-populated based on characteristics that were known about representative organizations at the time and augmented with a space to select and write in an “other” response where appropriate. Additional short answer questions were also created to capture more qualitative information. 

The second survey (administered in 2022–2023) included many of the questions used in the first survey, with additional questions to gather details relating to populations served and the incorporation of additional activities based on emerging areas of research and growing awareness of disparities, as well as about virtual TK models, given the rise in telehealth during the COVID-19 pandemic. These questions were integrated into the new TKC member applications (as of October 2017) because the information proved so valuable.

Each iteration of the survey was created by TKC staff, reviewed by at least three staff or members (including at least one of the coauthors of this article) to ensure questions were written clearly and included appropriate scope of questions and answers, and then tested by at least three staff or members to estimate the time to completion and assess validity. For the purpose of these surveys, TK models were defined as follows [15]:

- Mobile cart: portable and self-contained unit
- Pop-up (or modular) kitchen: temporary kitchen, assembled in a room or commercial kitchen
- Container/Pod: contained kitchen, transported via trailer or truck
- Built-in: dedicated, permanent kitchen space
- Virtual: remote kitchen space, accessed online

After necessary refinements were made based on the reviews, the request to complete each survey was sent to members via email and multiple reminder emails were sent for each survey to maximize completion rates. The primary contact person from each member organization (who was typically identified as being the program leader) was instructed to complete the survey on behalf of the organization. Administration of the two survey iterations is described below.

### 2.1. Iteration 1

Survey iteration 1 (Appendix A) was conducted between July 2017 and January 2019. (In this article, findings from this survey will be presented as representing 2019.) This time period was intentionally long to allow for targeted outreach to maximize responses (though 87% of responses were received in 2017). Total TKC membership included 39 organizations during this period. The survey consisted of 55 questions and was administered via Qualtrics. Categories of questions included reach and demographics; facilities, funding, staffing; curriculum elements; class format; priorities and shared aspirations; participants’ motivations for joining; financial considerations; and staffing models.

### 2.2. Iteration 2

Survey iteration 2 (Appendix B) was conducted between December 2022 and May 2023 with existing TKC members using the survey tool through Neon, which is a customer relationship management platform designed for nonprofit organizations that provides software for functions such as event management, fundraising, and supporter/member management. (In this paper, findings will be presented as representing 2023.) Total membership included 49 organizations during this period. To minimize the burden on respondents, TKC staff entered data that members had previously submitted through initial member applications and Qualtrics. The point of contact from each member organization was then asked to log in and either confirm or update its information, as needed. The survey included a total of 18 questions. To accommodate the need for assistance from members not familiar with Neon, the TKC membership services led three virtual conference calls in January and February 2023 to provide technical guidance on use of Neon. Also, membership staff manually created accounts for the contacts who experienced technical barriers to creating their new account in Neon so they could be prepared to complete the survey. Importantly, these steps did not influence responses in any way. Data from new member applications received via Google Forms in August 2022, which gathered identical descriptive data to the annual surveys, were included from six new members joining in 2023.

Data were downloaded from Qualtrics, Google Forms, and Neon and consolidated into one Microsoft Excel file so that responses to each survey could be summarized and analyzed. Sums and proportions (when appropriate) were calculated for responses to each question and these statistics were used to create a chart to visually present the findings. For questions that were asked in both 2019 and 2023, findings were presented in adjacent bars in the same graph. Responses from a subset of the survey questions are presented below.

## 3. Results

### 3.1. Survey Completion

TKC members in 2019 and 2023 that were asked to complete the surveys are listed in Appendix C. A total of 49 responses to the first survey iteration were received. Out of these 49 survey responses, 7 were excluded because the responders did not report their institutions and could not be identified (n = 42). In the three instances in which an organization submitted more than one set of responses, the latest complete sets of responses were retained for analysis, thus excluding three more responses (n = 39). Finally, at the time of the surveys, members were admitted to the TKC if they had a functional TK or plans to build one in the coming year. Given the goals of the survey, two responses were excluded because at the time of submission, their TKs were not yet operational, indicating that their responses about curriculum, funding, etc., would not be useful to describe the overall functions and impact of member organizations (n = 37; response rate of 94.9%). Sample sizes for individual questions that differed from this overall count are noted in figure captions below.

Survey 2 was sent to 43 existing members in December 2022 and 34 responses were collected by May 2023. Additionally, six new TKC members provided responses to the same questions in their applications submitted in August 2022, yielding a total of 40 responses (and an overall response rate of 81.6%) to the full set of survey questions.

All questions except for the number of participants served in the TK that year were marked as required. Therefore, responses were received from all members for all other questions and therefore were included in summaries below. Three respondents did not report the number of people served in their TKs and the six new members reported their participation for both in-person and virtual TKs combined.

**Figure 1:**
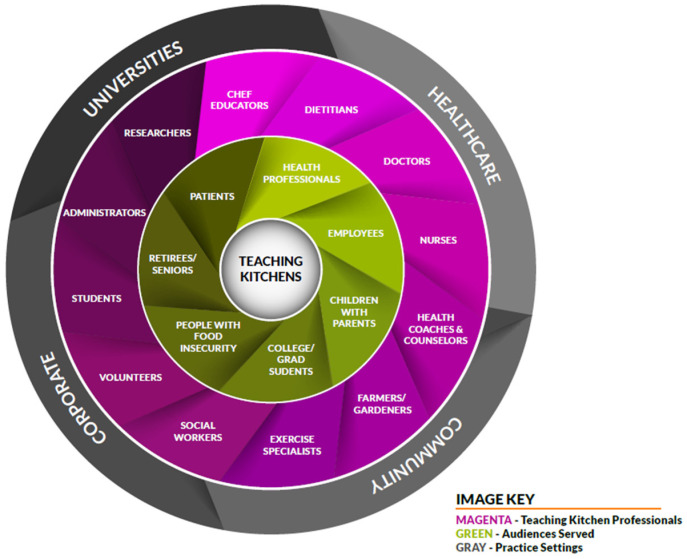
Landscape of TK programs.

Survey findings showed that members lead TK programs across a broad range of venues, audiences, staffing and funding models, and content matter. The general landscape of programs including audiences served, teaching kitchen professionals/program facilitators, and practice settings is shown in Figure 1; details about these findings are discussed in the subsections below.

### 3.2. Summary of Findings

**Figure 2:**
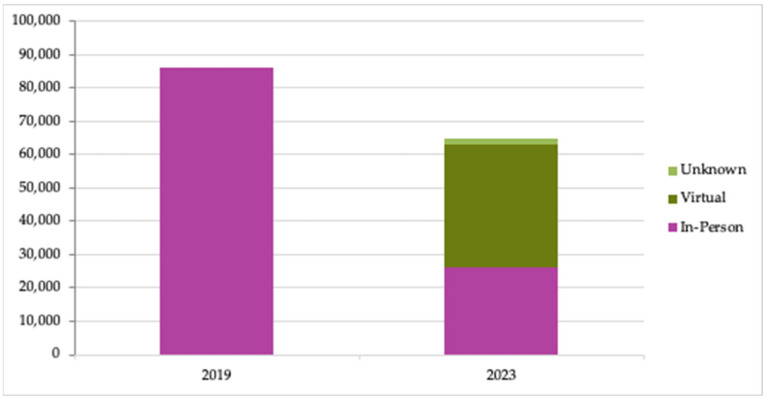
Number of participants served through TKC member organizations. (Number of members responding to surveys: N = 27 in 2019; N = 37 in 2023.)

Respondents estimated that a total of 86,237 and 64,912 participants were taught in TKs over the past year at the point of survey completion in 2019 and 2023, respectively (Figure 2). In 2023, respondents noted that they served more than half of their participants virtually. Members reflected in these counts serve audiences of very different sizes, however; one member with an active kitchen served only 12 participants in 2019. In 2019, more than one-third of participants were served through Oceania Cruises & Regent Seven Seas Cruises and in 2023, roughly one third of the participants were affiliated with the Veterans Affairs Healthy Teaching Kitchen programs.

These participant counts were derived by adding together the number of participants served by each organization over the prior year. While the surveys did not explicitly ask about how each organization counts and tracks its participants, previous discussions about this with a few member organizations revealed that they use a combination of several tactics: recruit and document patients from referring clinical departments through the electronic health record (EHR; either through individual or group codes); use a third-party registration platform for non-clinical classes; document general numbers of patients who were not referred by a clinical department in the EHR and identify the TK as a resource; maintain an additional spreadsheet of participant information within the TK’s department; use electronic forms to register and spreadsheets to track participants for certain community programs; or rely on external partner organizations for tracking.

#### 3.2.1. TK Setting(s)

**Figure 3:**
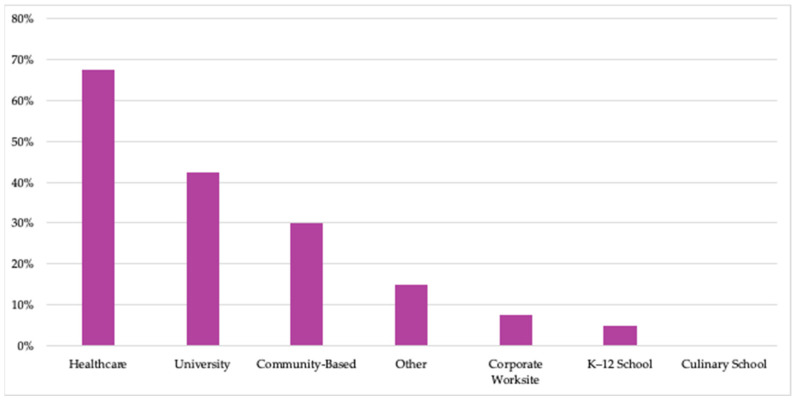
Percent of TKC member organizations that operated TKs in each practice setting in 2023. (Number of members responding to survey: N = 40.) Examples of “Other” responses included specific examples of healthcare, corporate, or community settings.

Healthcare was the most common type of practice setting among respondents (68%; Figure 3). Fifteen respondents (38%) identified as having more than one practice setting; these respondents reflected primarily universities with academic medical centers as well as two major companies that operate and serve diverse audiences either across the US or across the world.

#### 3.2.2. Audiences Served

**Figure 4:**
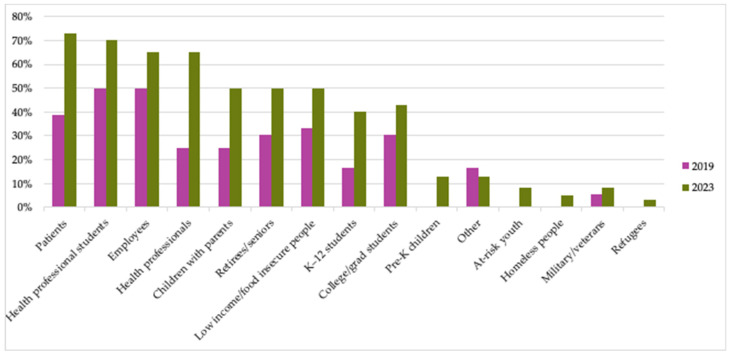
Percent of TKC member organizations that served each type of participant audience. (Number of members responding to surveys: N = 36 in 2019; N = 40 in 2023.) “Other” responses included formerly incarcerated/foster care/homeless youth, general community, and food-related media, grocery store, and food service buyers in 2019; and cancer survivors (×2), botanical garden members, and community members (×2) in 2023.

TKs serve a wide variety of audiences, with patient, health professional student, employee, and health professional audiences represented by more than half of respondents in 2023 (Figure 4). All but two respondents (95%) reported serving more than one type of audience and 25 respondents (63%) reported serving five or more types of audiences. Note that a shorter list of response options was presented in the 2019 survey, but there was sufficient overlap in response options to present the surveys’ responses together in Figure 4.

#### 3.2.3. TK Model(s)

**Figure 5:**
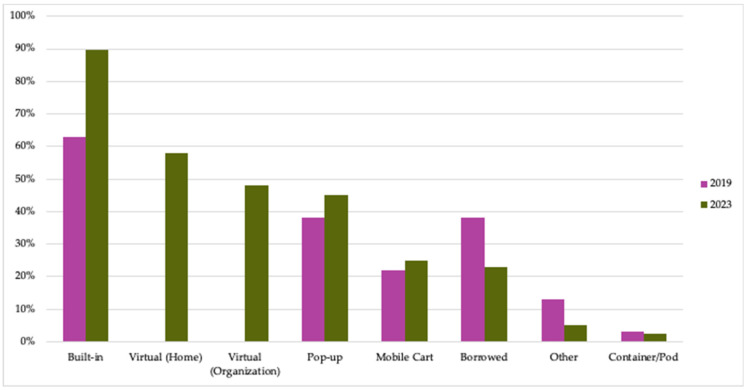
Percent of TKC member organizations that used each TK model. (Number of members responding to surveys: N = 31 in 2019; N = 40 in 2023.) Examples of “other” responses included community venues, dining halls, and outdoor kitchens in gardens.

TKC members lead programs in a wide variety of TK models (Figure 5). Built-in kitchens were used by the majority of members at both time periods. Further, in 2023, 90% of members used more than one model of TK to offer their programs—and some used as many as six different models. Virtual kitchens—whether based from instructors’ homes or institutions—were also used by the majority of members in 2023. (These were uncommon prior to the COVID-19 pandemic and were largely newly developed by TKC members in response to the necessary closure of in-person TKs in 2020.)

#### 3.2.4. Key Lines of Operations

**Figure 6:**
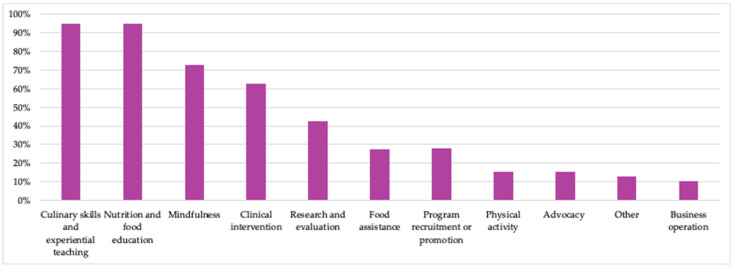
Key operations of TKC members’ programs in 2023. (Number of members responding to survey: N = 40.) Examples of “other” responses included gardening, instruction about collecting dietary histories, and engagement with employees.

Programs in TKs involve multiple lines of operations. Based on 2023 data, 95% of member organizations delivered culinary skills and experiential education and nutrition and food education through their TKs, with mindfulness incorporated in 73% (Figure 6). The majority of TKs also deliver clinical interventions in their TKs (63%); research and evaluation monitors progress in nearly half (43%). All members reported that they incorporate more than one operation into their TKs and 77% incorporate five or more operations.

#### 3.2.5. Professional Background of Team Member Who Manages or Directs the TK

**Figure 7:**
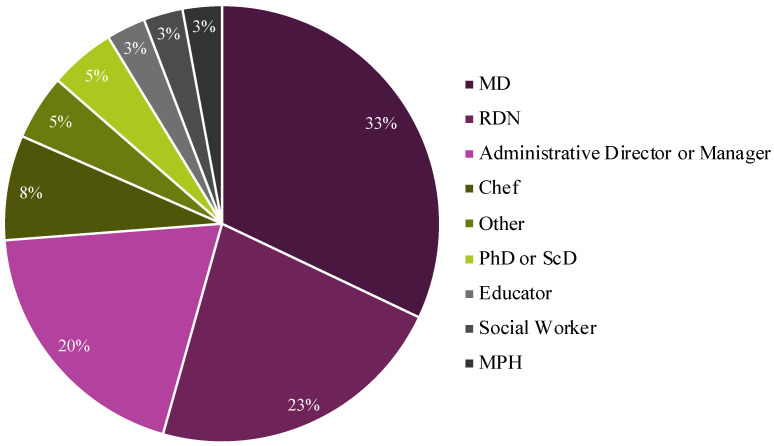
Type of professional who managed or directed the TK in 2023. (Number of member responding to survey: N = 40). The two “other” responses were chef/RDNs and the educator was described as having a Master of Food Service. Note that the percentages sum to slightly more than 100% due to rounding error.

Members indicated that a wide variety of professionals managed or directed their TKs in 2023 (Figure 7). The most common lead was a medical doctor (33%), followed by registered dietitian nutritionists (RDNs; 23%) and administrative directors or managers (23%).

#### 3.2.6. Team Member(s) Who Facilitate(s) TK Programming

**Figure 8:**
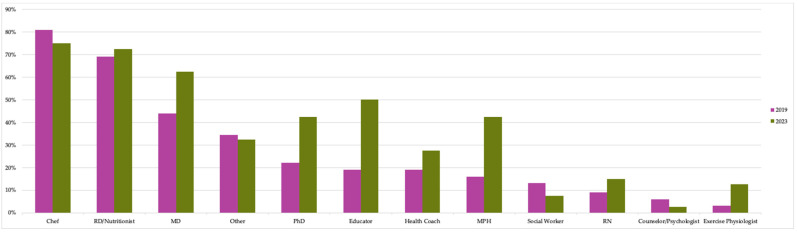
Percent of TKC member organizations whose instruction teams included each type of professional. (Number of members responding to surveys: N = 32 in 2019; N = 40 in 2023.) Examples of “Other Professionals” included Master of Library and Innovation Science, food scientists, health science graduate students, and physician assistants.

The most common professionals involved in facilitating TK programs across members at each point in time were chefs, followed closely by RDNs and medical doctors (Figure 8). In addition to the professionals or students/trainees shown in Figure 8, many members also relied on volunteers; however, it is unknown if respondents distinguished volunteers from other credentialed professionals in their responses.

Twenty-five percent also relied upon administrative assistants or directors for program operations. Further, responses to the 2023 survey offered additional types of instructors, such as medical students, gardeners, or farmers, that were not reflected—even as manual entries—in the original survey, which suggests a possible increase in the number of credentialed staff on each program team at the time of the second survey.

#### 3.2.7. Primary Funding Sources

**Figure 9:**
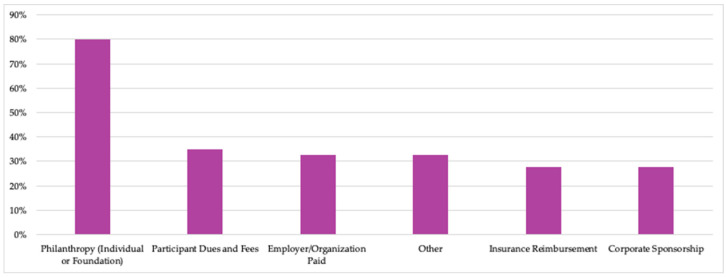
Percent of TKC member organizations that relied on each funding source in 2023. (Number of members responding to surveys: N = 40.) “Other” responses included licensing and training fees, operational revenue, institutional grants, and other internal funding mechanisms.

The most common funding source in both time periods was philanthropy; indeed, in 2023, 80% of members relied at least in part on philanthropic funding to operate their programs. The percentages of TKC members that used funding from various sources in 2023 are shown in Figure 9. (Because different response options were included in the 2019 survey, comparison between data from the two survey iterations in the same figure is not possible.) Notably, most members relied on multiple funding sources at both points in time: 81% and 74% of members relied on more than one source in 2019 and 2023, respectively.

Since the question about funding sources included three options reflecting some type of health insurance reimbursement (see question 18 in Appendix B), the number of respondents who selected each option were combined and deduplicated to produce Figure 9. However, among the total responses in 2023, 15% of respondents reported billing for shared medical appointments and 8% for medical nutrition therapy.

## 4. Discussion

This is the first description of a sample of diverse organizations with TKs that aim to improve culinary skills, nutrition, and food education for their programs’ participants. Findings also provide a basis for assessing the potential expansion and advancement of TKs. Decision-makers working in organizations outside of this sample but operating in the same academic, healthcare, business, or other setting can use these findings to consider implementation and expansion of TKs within their own organizations. At a time when Western countries are more burdened with mental and physical health conditions and spending more on healthcare than any other developed country, TKs in healthcare, private, and public settings present the prospect of providing long-needed solutions to sustainably promote a culture of health.

Survey results indicate the growth of TK organizations represented in this collaborative in both number and diversity over time. For example, they indicate an increase in credentialed professionals, who are likely needed to successfully perform the diverse set of operations implemented in each TK. This trend has thereby broadened the scope of programs reflected and the potential audiences served through TK programs as their teams continue to pursue the goal of expanding the movement and improving more lives through TKs. At the same time, trends in the fields of healthcare and food policy, which have changed and/or emerged since the TKC’s founding, position these organizations to chart new territory. Several figures show that healthcare-focused settings and audiences are most common, so it is possible that the most rapid spread of TKs is currently occurring in the healthcare sector based on the growing demand and opportunities for such evidence-based, food-focused health interventions described above.

Despite this growth, findings showed that the number of total participants served by this sample of organizations decreased between 2019 and 2023. This is likely due to the necessary closure of kitchens following the beginning of the COVID-19 pandemic. Then, as some TKs reopened gradually by 2023, they offered either or both in-person and virtual (while still interactive) classes.

Indeed, the rapid expansion of telehealth during the COVID-19 pandemic has presented new opportunities as well as challenges for culinary medicine. It has enabled more people to participate, the ability for participants to practice in their home kitchens, the delivery of multimedia content, and other benefits. Recent evaluations have shown virtual TK classes to be feasible, acceptable, and even effective in building skills and improving dietary quality [16,17,18]. But the pandemic also created challenges, such as the need to serve audiences both in person and online (often simultaneously) and the impediments to recreating and fostering community and friendships as participants learn together online. As more TK organizations return to in-person programming and/or implement hybrid formats, they would benefit from balancing the need to serve the most participants while also meeting the specific needs of their population(s) and utilizing their available resources. 

The TKC already offers structure, programming, and resources to member organizations that support them in their respective goals to improve their practice. Examples include myriad working groups to develop shared resources and a forthcoming multisite trial involving several members to investigate the benefits of TKs to the populations that they serve and then disseminate the findings [19]. In the future, the TKC could use these member survey findings to further forge successful partnerships across organizations and stakeholders based on their identified characteristics. Such partnerships can develop both within and outside of the Collaborative, including with the communities these TKs are intended to serve.

There are also many like-minded organizations with which this collaborative and other individual TK organizations can learn strategies to maximize impact. Several groups that are also focused on the fields of culinary medicine and/or culinary nutrition include the Culinary Nutrition Collaborative, Health Meets Food, Food and Culinary Professionals Dietetic Practice Group, and the American College of Lifestyle Medicine’s Culinary Medicine Member Interest Group [20,21,22,23]. While these groups serve specific groups of healthcare professionals, their models of education, training and/or their engagement in policy advocacy, and growing collaborations with the TKC offer opportunities to customize TKs for use across a wide spectrum of health professional communities. A wide variety of collaborative organizations aim to improve the US healthcare system more broadly; examples include the Primary Care Collaborative, Health Care Transformation Task Force, and the Health Equity Collaborative. While they do not focus on food and nutrition, these groups engage stakeholders from across sectors to improve healthcare quality and outcomes for patients and offer example tactics for TK organizations to collectively pursue similar goals.

Similarly, there are opportunities to further integrate within the growing FIM movement in the US. Organizations like the Food Is Medicine Collaborative and National Produce Prescription Collaborative are actively contributing to and building upon growing prioritization of food-based interventions throughout our healthcare system. These organizations are implementing tactics such as virtual and in-person training, campaigns to educate members of Congress, and accelerators and other technical support for budding programs to expand their presence and collective capacity to change policy and practice.

The FIM movement is making great progress as both public and private health plans in the US are increasingly covering food-based interventions (such as through Medicaid 1115 waivers, Medicare Advantage [MA] supplemental benefits, and other mechanisms) [24]. Similarly, alternative payment models are presenting new funding mechanisms for FIM interventions, including TKs. The Centers for Medicare & Medicaid Services Innovation Center’s new Accountable Care Organization Realizing Equity, Access, and Community Health (ACO REACH) model encourages coordination across healthcare providers to deliver high-quality, equitable care and gives providers more flexibility to use dollars in ways that meet patients’ needs—such as improved access to healthy groceries and building skills to cook them. Similarly, the recently extended MA Value-Based Insurance Design model will be modified to further address patients’ health-related social needs, including through enhanced access to healthy groceries and nutrition professionals. While TKs are a fundamental component of the FIM spectrum of interventions [25], they are not always recognized as such in practice [4]. Though such new payment models are not reflected in the two surveys administered thus far, they present opportunities for TKs across the US to expand and secure sustainable funding and their application could be explored in future research among TKs.

Multidisciplinary teams are fundamental to successful TK program implementation and participant engagement—and their prevalence is indicated through these survey findings. While some training and continuing education opportunities for medical students, residents, RDNs, and chefs already exist, other fields that play important roles in ensuring access to food and safe use of equipment—such as social work and occupational therapy—do not necessarily have access to the same opportunities and are not as frequently included in care teams. Participating in a collaborative of TKs like the TKC positions members well to lead and grow interprofessional education and training for such groups to ensure all key team members can be included in TK programs across the country. Further, the TKC can pursue new areas of practice to most effectively apply both its diverse members’ skills and passions and to respond to the external environment—and then evaluate the impact of their TKs through both prospective and retrospective studies.

Using the TKC membership as a convenience sample to describe TKs may have introduced sources of bias that skewed the characteristics quantified above. First, the healthcare expertise of the TKC’s leadership may have contributed to the observed greater number of healthcare-focused settings and audiences. Additionally, the TKC is a dues-collecting organization, so smaller, community-based organizations may have lesser ability than the larger organizations focused on healthcare and academia to be able to afford to join. Not all TK organizations have the capability to distinguish unique participants but rather track total encounters, which may have led to an inflated total estimate of participants served through this TK sample.

Further, the two surveys did not include the exact same set of questions, were not administered at an equal cadence, and were not administered through the same electronic platform. These differences in methods create variability that may prevent direct comparisons between surveys. However, the survey questions reflected the same themes, and most can be used to inform general descriptions about recently launched TKs in the US and select international settings.

To continue this research, a comparable survey should be conducted every few years to understand growth in the scope and impact of TKs as well as to continue to understand progress toward organizational goals. Additional opportunities for future research could include conducting member interviews and analyzing feedback for themes that can provide more detailed responses or surveys of other audiences served by TKC member activities to understand what non-member organizations working in culinary medicine are accomplishing—and what they could better accomplish through joining such an innovative, mission-driven organization.

Additional research could help to better understand which audiences are served best by TKs that have each model and program structure, providing information to inform appropriate modifications to better reach those audiences in the future. While teaching teams can be most effective when they include experts from multiple disciplines, future research could investigate and identify which particular skills can be taught most effectively—and possibly even which outcomes can be achieved—when certain types of expertise are reflected in the teaching/leadership team to help organizations make informed hiring decisions. Finally, future research can investigate how TKs regularly seek feedback from their own participants to better understand their needs and interests and then use that feedback to inform process improvements. Such information can also be aggregated and shared to provide helpful guidance for other TKs to ensure they are providing programming and resources best tailored to their audiences; it can also be used to better understand which audiences who may need to access their programs are not yet able to do so.

Findings from the first two surveys conducted of TKC member organizations show the wide diversity in current models and operations of TKs, as well as possible indications of changes over time, as these TKs have had to respond to external factors to best sustain operations, serve their participants, and optimize their impact. Findings can inform future strategies for TK organizations—whether part of the TKC or otherwise—as well as enhance understanding of the scope and impact of TKs among external stakeholders who may be in positions to help further advance the field.

## Data Availability

The data used and/or analyzed during the current study are available upon request from the corresponding author. The data are not publicly available because they contain proprietary and/or identifiable information and lack access to a public database.

## Appendix A. First TKC Member Survey Administered through Qualtrics

Thank you for taking the Teaching Kitchen Collaborative’s member survey. It has been about two years since the inception of the Collaborative, therefore we are seeking to understand the progress of TKC member organizations, the Collaborative’s collective impact, and how we can continue to improve the Collaborative.

This survey will take approximately 20 min. Please engage those TKC members within your organization with relevant knowledge to complete this survey. Please complete by Tuesday, 1 August.

1. Please provide the full name of your organization and affiliated center.
2. Who is the primary contact completing this form?
3. Have there been any significant changes to your teaching kitchen(s) and TK related programs since you joined the Teaching Kitchen Collaborative (e.g., renovations, opening of new kitchen, etc.)?

The following questions pertain to your teaching kitchen teaching team.

4. How many people (full or part-time) are on your teaching team?
  • 1
  • 2
  • 3
  • 4
  • 5 and up

For the following questions, please fill in credentials for EACH person on your teaching team.

5. Person #1: What type of credentials does your instructor have? (select all that apply as some may have multiple credentials)
  • Chef/culinary training (e.g., CEC, AAC)
  • Medical doctor
  • Dietitian (e.g., RD, CSSD, RDN, LD)
  • Nurse (e.g., RD, APRN, FNP)
  • Master of Public Health
  • Social work (e.g., MSW)
  • Counseling/psychology
  • Educator (e.g., Certified Diabetes Educator, Master of Education)
  • Health Coach
  • Exercise physiologist
  • No specific credential/volunteer
  • PhD or ScD
  • Administrative assistant/administrator
  • Other [write in] ____________________
6. Person #2: What type of credentials does your instructor have? (select all that apply as some may have multiple credentials)
  • Chef/culinary training (e.g., CEC, AAC)
  • Medical doctor
  • Dietitian (e.g., RD, CSSD, RDN, LD)
  • Nurse (e.g., RD, APRN, FNP)
  • Master of Public Health
  • Social work (e.g., MSW)
  • Counseling/psychology
  • Educator (e.g., Certified Diabetes Educator, Master of Education)
  • Health Coach
  • Exercise physiologist
  • No specific credential/volunteer
  • PhD or ScD
  • Administrative assistant/administrator
  • Other [write in] ____________________
7. Person #3: What type of credentials does your instructor have? (select all that apply as some may have multiple credentials)
  • Chef/culinary training (e.g., CEC, AAC)
  • Medical doctor
  • Dietitian (e.g., RD, CSSD, RDN, LD)
  • Nurse (e.g., RD, APRN, FNP)
  • Master of Public Health
  • Social work (e.g., MSW)
  • Counseling/psychology
  • Educator (e.g., Certified Diabetes Educator, Master of Education)
  • Health Coach
  • Exercise physiologist
  • No specific credential/volunteer
  • PhD or ScD
  • Administrative assistant/administrator
  • Other [write in] ____________________
8. Person #4: What type of credentials does your instructor have? (select all that apply as some may have multiple credentials)
  • Chef/culinary training (e.g., CEC, AAC)
  • Medical doctor
  • Dietitian (e.g., RD, CSSD, RDN, LD)
  • Nurse (e.g., RD, APRN, FNP)
  • Master of Public Health
  • Social work (e.g., MSW)
  • Counseling/psychology
  • Educator (e.g., Certified Diabetes Educator, Master of Education)
  • Health Coach
  • Exercise physiologist
  • No specific credential/volunteer
  • PhD or ScD
  • Administrative assistant/administrator
  • Other [write in] ____________________
9. Person #5: What type of credentials does your instructor have? (select all that apply as some may have multiple credentials)
  • Chef/culinary training (e.g., CEC, AAC)
  • Medical doctor
  • Dietitian (e.g., RD, CSSD, RDN, LD)
  • Nurse (e.g., RD, APRN, FNP)
  • Master of Public Health
  • Social work (e.g., MSW)
  • Counseling/psychology
  • Educator (e.g., Certified Diabetes Educator, Master of Education)
  • Health Coach
  • Exercise physiologist
  • No specific credential/volunteer
  • PhD or ScD
  • Administrative assistant/administrator
  • Other [write in] ____________________
10. What percent of your entire instructional team is full-time, part-time (paid), and volunteer in terms of their commitment to the TK program? [must add to 100%]
  ______ Full Time
  ______ Part Time (Paid)
  ______ Volunteer

The following questions pertain to your teaching kitchen curriculum.

To what extent do you incorporate the following topics into your teaching kitchen classes, if applicable?

11. Culinary Skills

	**Taught in All Classes**	**Taught in Some Classes**	**Not Taught**
Kitchen set up and safety	🔾	🔾	🔾
Basic cooking/food preparation skills	🔾	🔾	🔾
Healthy cooking techniques	🔾	🔾	🔾
Meal planning and food shopping strategies	🔾	🔾	🔾
Guided grocery store and/or farm/farmers’ market tour	🔾	🔾	🔾

12. Are there any other major topics within Culinary Skills that you cover? Please describe.
13. Nutrition & Health

	**Taught in All Classes**	**Taught in Some Classes**	**Not Taught**
General guidelines for healthy dietary patterns	🔾	🔾	🔾
Recommendations for specific foods/nutrients	🔾	🔾	🔾
Disease-specific recommendations	🔾	🔾	🔾
Food systems, sustainability, waste considerations	🔾	🔾	🔾

14. Are there any other major topics within Nutrition & Health that you cover? Please describe.
15. Exercise & Movement

	**Taught in All Classes**	**Taught in Some Classes**	**Not Taught**
General guidelines for increased physical activity	🔾	🔾	🔾
Guided exercises/activity bursts during class session(s)	🔾	🔾	🔾

16. Are there any other major topics within Exercise & Movement that you cover? Please describe.
17. Mindfulness

	**Taught in All Classes**	**Taught in Some Classes**	**Not Taught**
Definition and benefits of mindfulness as applied to eating, cooking, exercising, sleep, etc.	🔾	🔾	🔾
Guided mindfulness exercises during class session(s)	🔾	🔾	🔾

18. Are there any other major topics within Mindfulness that you cover? Please describe.
19. Health Coaching & Behavior Change

	**Taught in All Classes**	**Taught in Some Classes**	**Not Taught**
Individual health coaching with program participants	🔾	🔾	🔾
Facilitated group discussion	🔾	🔾	🔾
Follow-up sessions after intervention period	🔾	🔾	🔾

20. Are there any other major topics within Health Coaching & Behavior Change that you cover? Please describe.
21. Are there any other major topics outside of Culinary, Nutrition & Health, Exercise & Movement, Mindfulness, and Health Coaching & Behavior Change that you cover? Please describe.
22. Do you use a standard teaching kitchen curriculum that you license or purchased?
  • We currently use a curriculum that we purchased or license. [skip questions 24 and 25]
  • We considered purchasing or licensing a curriculum, but decided not to [skip to questions 23 and 25]
  • We have not purchased or licensed a curriculum [skip questions 23 and 24]
23. Which curriculum did you purchase/license and why?
24. Which curriculum did you consider purchasing or licensing and why did you decide not to go forward with it?
25. Please describe what curriculum you use.
26. How often are participants involved in the following activities during your teaching kitchen program?

	**In Every Class**	**In Some Classes**	**Never**
Watching lectures	🔾	🔾	🔾
Viewing cooking demonstrations	🔾	🔾	🔾
Performing hands-on cooking	🔾	🔾	🔾
Tasting	🔾	🔾	🔾
Homework/home assignments	🔾	🔾	🔾
Other [please describe]	🔾	🔾	🔾

The following questions pertain to your teaching kitchen facilities.

27. Please specify the type of kitchen facility or facilities you employ (mark all that apply):
  • Mobile Cart: Portable and self-contained unit; can be as simple as a burner plate on a cart or a fully furnished mobile unit
  • Pop-Up/Modular Kitchen: Temporary kitchen for short term; pieced together with cooktops, cutting boards, kitchenware, etc.; either with or without a dedicated space
  • Container/pod: Contained kitchen, either transported via trailer or truck
  • Built-In: Dedicated, permanent kitchen space
  • Borrowed: Borrowing kitchen/space at another organization’s facility (e.g., local culinary institute, community center, school) [please describe] ____________________
  • Other [please describe] ____________________
28. What approximate level of up-front investment was made in your kitchen facility/facilities? Give your best guess estimate.
  • Under $1000
  • $1000–$10,000
  • $10,000–$50,000
  • $50,000–$100,000
  • $100,000–$200,000
  • $200,000–$300,000
  • $300,000–$400,000
  • $400,000–$500,000
  • Over $500,000
29. On an annual basis, approximately how much does it cost to run your teaching kitchen and associated programs (including staff, programing, facilities upkeep, etc.). Give your best guess estimate.
  • Under $5000
  • $5000–$20,000
  • $20,000–$35,000
  • $35,000–$50,000
  • Over $50,000
30. Approximately what percentage of the total cost of running your teaching kitchen program is in each category? [categories must sum to 100%]
  ______ Staff salaries
  ______ Programing (e.g., curriculum development, purchase)
  ______ Incremental class supplies (e.g., food, disposable supplies, printed materials, etc.)
  ______ Ongoing facilities investments and maintenance (e.g., new equipment)
  ______ Other [please describe]
31. Approximately what are the main sources of funding for your teaching kitchen programs? [categories must sum to 100%]
  ______ Participant dues and fees
  ______ Philanthropy (individual donations, foundation grants)
  ______ Corporate sponsorships
  ______ Endowment
  ______ Other [please describe]
32. Have you found any strategies to be particularly effective in securing funding for your teaching kitchen program? If so, please describe.

The following questions pertain to measuring the Collaborative’s collective impact.

33. Please specify the primary audience of focus for your teaching kitchen classes (check all that apply).
  • Employees
  • Patients
  • Children with their parents
  • Students (K–12)
  • College/graduate students
  • Health professionals students (medical students, dietetic interns, etc.)
  • Health professionals (MD, RD, RN, etc.)
  • Retirees/senior citizens
  • Military
  • Low-income/food insecure
  • Other [please describe] ____________________
34. What is the primary reason your participants attend teaching kitchen classes? (check all that apply).
  • As a preventive health measure
  • To address health issues
  • To support family members (children, spouses, parents) in achieving health goals
  • Due to a health professional recommendation or referral
  • To obtain a financial incentive provided by employer or insurer (e.g., HSA money, health care premium breaks, etc.)
  • For fun or to learn a new skill
  • Other [please describe] ____________________
35. Estimate how many total participants have participated in the last 12 months?
36. Estimate how many total participants have participated since you first joined the Teaching Kitchen Collaborative?
37. Estimate what percentage of your participants have joined each type of teaching kitchen program [must sum to 100%]?
  ______ One-off classes
  ______ Mini series (2–4 classes)
  ______ Longer series (5–9 classes)
  ______ Transformation program (10+ classes)
38. What type of data, if any, do you collect on participants?
  • Pre/post self-reported data on knowledge and/or skills
  • Feedback on classes and recipes
  • General descriptive data (e.g., age, gender, race)
  • Medical history (e.g., diabetes, hypertension, heart disease)
  • Body measurements (height, weight)
  • Biometric data (e.g., lipid panel, blood sugar)
  • We do not collect data at this time
  • Other [please describe] ____________________
39. Have you or your team collaborated with any other TKC member organizations outside of organized TKC meetings and calls (e.g., sharing/brainstorming program strategies, curriculum, and/or other resources)? If so, which member organizations have you collaborated with and in what capacities?
  • No
  • Yes [please describe] ____________________
40. Have ideas from other collaborative members meaningfully impacted your teaching kitchen program? If so, please share an example or two.

The following questions pertain to how we can improve the Collaborative. Please answer as honestly as possible!

41. On a scale from 0–10, how likely are you to recommend the Teaching Kitchen Collaborative to other similar organizations to yours?
  • 0
  • 1
  • 2
  • 3
  • 4
  • 5
  • 6
  • 7
  • 8
  • 9
  • 10
42. What has the Collaborative been most useful for?
43. What has the Collaborative been least useful for?
44. Rate the value of your TKC membership to your organization.
  • Extremely valuable
  • Very valuable
  • Moderately valuable
  • Slightly valuable
  • Not at all valuable

The Teaching Kitchen Collaborative is considering investing in the following initiatives:

(1) Expanded Best Practices: Additional documents and webinars with best practices (e.g., facility and curriculum design)
(2) Cooking Videos: A library of cooking videos
(3) Core Curriculum: A complete teaching kitchen curriculum
(4) TK Starter Kit: A “turnkey” guide used for end-to-end creation of a TK (including facility design, curriculum, staffing etc.)
(5) Research Consulting: More assistance on research (e.g., consulting on measuring program efficacy, research conferences, webinars)
(6) Data Platform: Data platform accessible by members, used to track outcomes
(7) Outcomes Research: Large, multi-site projects to demonstrate improvement in behaviors, clinical outcomes and cost
(8) Standard Setting: Creating accreditation standards for TKs including facilities, trainers, curricula
(9) Train the Trainer: Training sessions (in person or online) for TK facilitators

Please use these descriptions to answer the following questions.

45. What, in your team’s opinion, are the three most important initiatives for the Teaching Kitchen Collaborative to focus on over the next 24 months? Please rank only your top 3 areas of focus.

	**(1) Best Practices**	**(2) Cooking Videos**	**(3) Core Curriculum**	**(4) TK Starter Kit**	**(5) Research Consulting**	**(6) Data Platform**	**(7) Outcomes Research**	**(8) Standard Setting**	**(9) Train the Trainer**
Top Priority	🔾	🔾	🔾	🔾	🔾	🔾	🔾	🔾	🔾
Second Priority	🔾	🔾	🔾	🔾	🔾	🔾	🔾	🔾	🔾
Third Priority	🔾	🔾	🔾	🔾	🔾	🔾	🔾	🔾	🔾

46. Before finishing this survey, is there anything else we should know in order to make the Collaborative increasingly valuable for your organization?

## Appendix B. Second TKC Member Survey Administered through Neon

1. Full Name
2. Company/Organization Name
3. Email
4. Phone
5. Address
6. In which type of setting is your teaching kitchen? Mark all that apply.
  • Community-Based
  • Corporate Worksite
  • Culinary School
  • Healthcare
  • K–12 School
  • University
  • Other [please describe]
7. What is the name of your teaching kitchen or program?
8. How many teaching kitchens does your organization operate?
9. Which describes your teaching kitchen facility(ies)? Mark all that apply.
  • Borrowed
  • Built-in
  • Container/Pod
  • Mobile Cart
  • Pop-up
  • Virtual (organization teaching kitchen)
  • Virtual (home kitchen)
  • Other [please describe]
10. What populations do you work with in your teaching kitchen? Mark all that apply.
  - At-risk youth
  - Children with parents
  - K–12 students
  - Pre-K children
  - College/Graduate students
  - Employees
  - Health professional students
  - Health professionals
  - Homeless people
  - Low-income/Food insecure people
  - Military/Veterans
  - Patients
  - Refugees
  - Retirees/Seniors
  - Other [please describe]

11. What health and social factors does your teaching kitchen address? Mark all that apply.
  • Addiction recovery
  • Bariatric surgery
  • Brain health
  • Cancer
  • Chronic pain
  • Cardiovascular disease
  • Diabetes
  • Food access
  • Maternal and child health
  • Mental health
  • Optimizing health & wellbeing
  • Other [please describe]
12. What fields, programs, or therapies are involved in your teaching kitchen? Mark all that apply.
  • Diabetes Prevention Program
  • Educating health professionals
  • Fresh food farmacy
  • Integrative medicine
  • Lifestyle medicine
  • Gardening, local farms, & agriculture
  • Medical nutrition therapy
  • Medically tailored meals
  • Produce prescription
  • Shared medical appointments
  • Other [please describe]
13. Which is/are a key function(s) of your teaching kitchen? Mark all that apply.
  • Advocacy
  • Business operation
  • Clinical intervention
  • Culinary skills and experiential teaching
  • Food assistance
  • Nutrition and food education
  • Mindfulness
  • Physical activity
  • Program recruitment or promotion
  • Research and evaluation
  • Other [please describe]
14. How many professionals are included in your core teaching kitchen team, meaning involved in most essential operations of the TK?
15. Now consider the wider collaboration in your organization on the TK. How many professionals are included in your extended teaching kitchen team, meaning occasional instructors, volunteers, or researchers?
16. What type of professional is the overall teaching kitchen lead?
  • Administrative assistant
  • Administrative director or manager
  • Chef
  • Counselor/psychologist
  • Educator
  • Exercise physiologist
  • Health coach
  • MD
  • MPH
  • Nurse
  • PhD or ScD
  • Registered dietitian
  • Social worker
  • Volunteer
  • Other [please describe]
17. Which other types of professionals facilitate classes in the teaching kitchen? Mark all that apply.
  • Administrative assistant
  • Administrative director or manager
  • Chef
  • Counselor/psychologist
  • Educator
  • Exercise physiologist
  • Health coach
  • MD
  • MPH
  • Nurse
  • PhD or ScD
  • Registered dietitian
  • Social worker
  • Volunteer
  • Other [please describe]
18. Which sources contribute to funding your teaching kitchen? Mark all that apply.
  • Corporate sponsorship
  • Diabetes Prevention Program
  • Employer/organization paid (department chargeback)
  • Insurance reimbursement
  • Medical nutrition therapy
  • Participant dues and fees (participant paid)
  • Philanthropy (individual or foundation)
  • Shared medical appointments
  • Other
19. What primary language(s) other than English do your participants speak?
20. In what language(s) do you teach classes in your teaching kitchen?
  • English
  • Chinese
  • French
  • Spanish
  • Tagalog
  • Vietnamese
  • Other [please specify]
21. How many people participated in your teaching kitchen in person in 2022?
22. How many people participated in your teaching kitchen virtually in 2022?
23. Does this refer to the unique number of participants or total number of visits (including repeat visitors)?
  • Unique number of participants
  • Total number of visits including repeats
  • Not sure

## Appendix C. List of TKC Member Organizations at the Time Each Survey Iteration Was Conducted

**2019**	**2023**
1440 Multiversity Barilla & Barilla Center for Food & Nutrition Foundation Boston Medical Center, Nutrition Resource Center’s Demonstration Kitchen and Preventive Food Pantry CancerScan (formerly Campus for H) Cleveland Clinic Compass Group, North America Dartmouth-Hitchcock Culinary Medical Program EHM Senior Solutions FamilyCook Productions Free Library of Philadelphia Culinary Literacy Center Google, Inc. Hackensack Meridian Health Harvard University Kaiser Permanente San Francisco Medical Center MaineGeneral Health Prevention and Healthy Living Medstar Health Northeastern Dining Northwell Health Northwestern University (Osher Center for Integrative Medicine) Oceania Cruises & Regent Seven Seas Cruises Palo Alto Medical Foundation Princeton University Providence Milwaukie Hospital Stanford University (Residential & Dining Enterprises) Turner Farm Inc. in collaboration with University of Cincinnati (Academic Health Center & College of Medicine, Center for Integrative Health and Wellness) University of California, Berkeley (College of Natural Resources; Health Services) University of California, Los Angeles (Chancellor Block’s Healthy Campus Initiative & UCLA Dining) University of California, San Francisco (Osher Center for Integrative Medicine) University of Michigan University of Minnesota (Center for Spirituality & Healing) University of New Hampshire University of Southern California (Keck School of Medicine) University of South Carolina School of Medicine Greenville University of Texas Health Science Center at Houston (UTHealth School of Public Health) University of Texas Medical Branch and Osher Lifelong Learning Institute, Galveston University of Vermont Medical Center Vanderbilt University Medical Center (Osher Center for Integrative Medicine and Center for Biomedical Ethics & Society) Veterans Health Administration Healthy Teaching Kitchen Program YMCA of Greater Pittsburgh-Sampson Family Branch	1440 Multiversity Alberta Health Services/University of Calgary Apples to Zucchini Cooking School Banyan Barilla & Barilla Center for Food & Nutrition Foundation Bon Secours Richmond Boston Medical Center, Nutrition Resource Center’s Demonstration Kitchen and Preventive Food Pantry CancerScan Case Western Reserve University Children’s Healthcare of Atlanta Cincinnati Hills Christian Academy Cleveland Clinic Compass Group, North America Culinary Medicine Germany e.V. Dartmouth-Hitchcock Culinary Medical Program Emory University Lifestyle Medicine & Wellness FamilyCook Productions Free Library of Philadelphia Culinary Literacy Center Google, Inc. Griffin Health Hackensack Meridian Health Kaiser Permanente Bernard J. Tyson School of Medicine Kaiser Permanente San Francisco Medical Center MaineGeneral Health Prevention and Healthy Living Marshall University/Huntington Kitchen Medstar Health Moffitt Cancer Center Northeastern Dining Northwell Health Northwestern University (Osher Center for Integrative Medicine) Phipps Conservatory and Botanical Gardens Presbyterian Healthcare Services Providence Milwaukie Hospital Spartanburg Regional Healthcare System Spaulding Rehabilitation Network Turner Farm Inc. in collaboration with University of Cincinnati (Academic Health Center & College of Medicine, Center for Integrative Health and Wellness) University of British Columbia University of California, Berkeley (College of Natural Resources; Health Services) University of California, Irvine (Susan Samueli Integrative Health Institute) University of California, Los Angeles (Chancellor Block’s Healthy Campus Initiative & UCLA Dining) University of Minnesota (Center for Spirituality & Healing) University of South Alabama University of Southern California (Keck School of Medicine) University of Texas Health Science Center at Houston (UTHealth School of Public Health) (The Culinary Medicine Program at) University of Texas Southwestern University of Utah Center for Community Nutrition and Osher Center University of Vermont Medical Center Veterans Health Administration Healthy Teaching Kitchen Program YMCA of Greater Pittsburgh-Sampson Family Branch
